# CircRNA circ_0072995 promotes the progression of epithelial ovarian cancer by modulating miR-147a/CDK6 axis

**DOI:** 10.18632/aging.103668

**Published:** 2020-09-02

**Authors:** Jin Ding, Qingwei Wang, Nan Guo, Hao Wang, He Chen, Guantai Ni, Peiling Li

**Affiliations:** 1 Department of Obstetrics and Gynecology, The Second Affiliated Hospital of Harbin Medical University, Harbin 150086, Heilongjiang, P.R. China; 2 Department of Obstetrics and Gynecology, First Affiliated Hospital of Wannan Medical College, Wuhu 241000, Anhui, P.R. China; 3 Department of Gynecology, International Peace Maternity and Child Health Hospital, Shanghai Jiao Tong University School of Medicine, Shanghai 310000, P.R. China; 4 Department of Obstetrics and Gynecology, Shenzhen Second People’s Hospital, First Hospital of Shenzhen University, Shenzhen 518000, Guangzhou, P.R. China

**Keywords:** circular RNA, circ_0072995, epithelial ovarian cancer, miR-147a, CDK6

## Abstract

Background: Increasing evidence has indicated that circular RNAs (circRNAs) play vital roles in modulating tumor progression. However, regulatory roles and underlying mechanisms of circRNA circ_0072995 in epithelial ovarian cancer (EOC) are not well characterized.

Results: Circ_0072995 was up regulated in EOC afflicted tissues and cell lines (HO8910 and A2780), and was mainly located in the cytoplasm. The expression of circ_0072995 was associated with the pathological grade of EOC for respective patients. Functional experiments revealed that circ_0072995 promoted EOC cell proliferation, migration, induced apoptosis, as well as enhanced tumorigenesis in vivo. Mechanistic analyses indicated that circ_0072995 may have acted as a sponge of miR-147a such as to relieve repressive effects of miR-147a upon its target CDK6.

Conclusions: Our results revealed that circ_0072995 promoted EOC progression through the circ_0072995/miR-147a/CDK6 axis and may represent a strategy for treatment of EOC afflicted patients.

Methods: Expression of circ_0072995 was evaluated in 40 EOC tissue samples and cell lines by qRT-PCR. The location of circ_0072995 was determined via nuclear-cytoplasmic fractionation. A series of functional experiments facilitated determinations of effects of circ_0072995 on EOC progression in vitro, and in vivo. Underlying mechanisms and influence of circ_0072995 on EOC were confirmed by bioinformatic analyses, luciferase reporter assays, qRT-PCR, and Western blotting.

## INTRODUCTION

Ovarian cancer (OC) is one of the most fatal types of malignant tumors that threatens female health and is the second deadliest type of female gynecologic cancer [1]. In 2020, it is approximated that 21,750 new cases of OC and 13,940 OC caused deaths will result among females in the U.S.A. [2]. Epithelial ovarian cancer (EOC) is the main pathological type of ovarian cancer and progresses insidiously and rapidly as well as is prone to extensive invasion and metastasis [3, 4]. Despite these impacts and known dynamics, the lack of effective diagnostic methods to detect EOC when present in its early stages has clearly been linked to worse prognoses, a higher risk of distant recurrence, and a relatively poor 5-year survival rate for EOC afflicted patients which falls below 45 % [5]. Therefore, it is of particular urgency to uncover novel molecular targets for improved therapy and treatment outcomes for EOC afflicted patients.

Circular RNAs (circRNAs) have been used extensively in clinical experiments and are of great potential as biomarkers for detection of disease [6, 7], and could hold some promise for improving EOC diagnosis and treatment. Due to their special structure which consists of a covalently closed continuous loop with neither 5’-3’ polarity nor a polyadenylated tail, circRNAs are protected against the effects of RNA enzyme based degradation and can be used as a class of stable molecular marker [8]. With the development of RNA-seq and bioinformatics based technologies, recent studies have elucidated the potential utilization of circRNAs for the early detection and prognostication of some cancer types, including lung adenocarcinomas [9], breast cancers [10], and osteosarcoma [11]. However, the dynamics of regulatory roles and underlying mechanisms of most circRNAs in pathogenesis and progression of EOC are not well understood and have not been examined in such detail comparatively.

Generally, circRNAs act as microRNA (miRNA) sponges, competing for miRNA binding and affecting miRNA function [12, 13]. Some circRNAs can even regulate disease progression by binding protein [14], through N6-methyladenosine modification [15], and by encoding protein [16]. Further, it has been suggested that circRNAs may be closely associated with tumor occurrence and progression, and might be used as novel biomarkers and therapeutic targets for cancer diagnosis and treatment. For example, Hsa_circ_0072995 is located at chr5:73069679-73076570 with a 435 bp spliced length, derived from exon 5-7 of the ARHGEF28 gene and may have some potential for such applications, but is yet to be examined. In previous studies, our team found that circ_0072995 is overexpressed in epithelial ovarian cancer afflicted tissues. Previous research has indicated that circ_0072995 promoted migration and invasion in breast cancer cells [17], thus, we speculated that circ_0072995 might play an important role in the regulation of development of EOC. In support of this hypothesis, a previously completed series of functional experiments illustrated the crucial role of circ_0072995 upon the dynamics of ovarian cancer tumorigenesis. Thus, we furthermore also sought to examine the interactions of circ_0072995 with the miR-147a/CDK6 axis. We hoped that our results would provide valuable information furthering prevention strategies and treatment outcomes for EOC.

## RESULTS

### Circ_0072995 was upregulated in EOC tissues and cell lines, and was associated with tumor pathology

**Figure 1:**
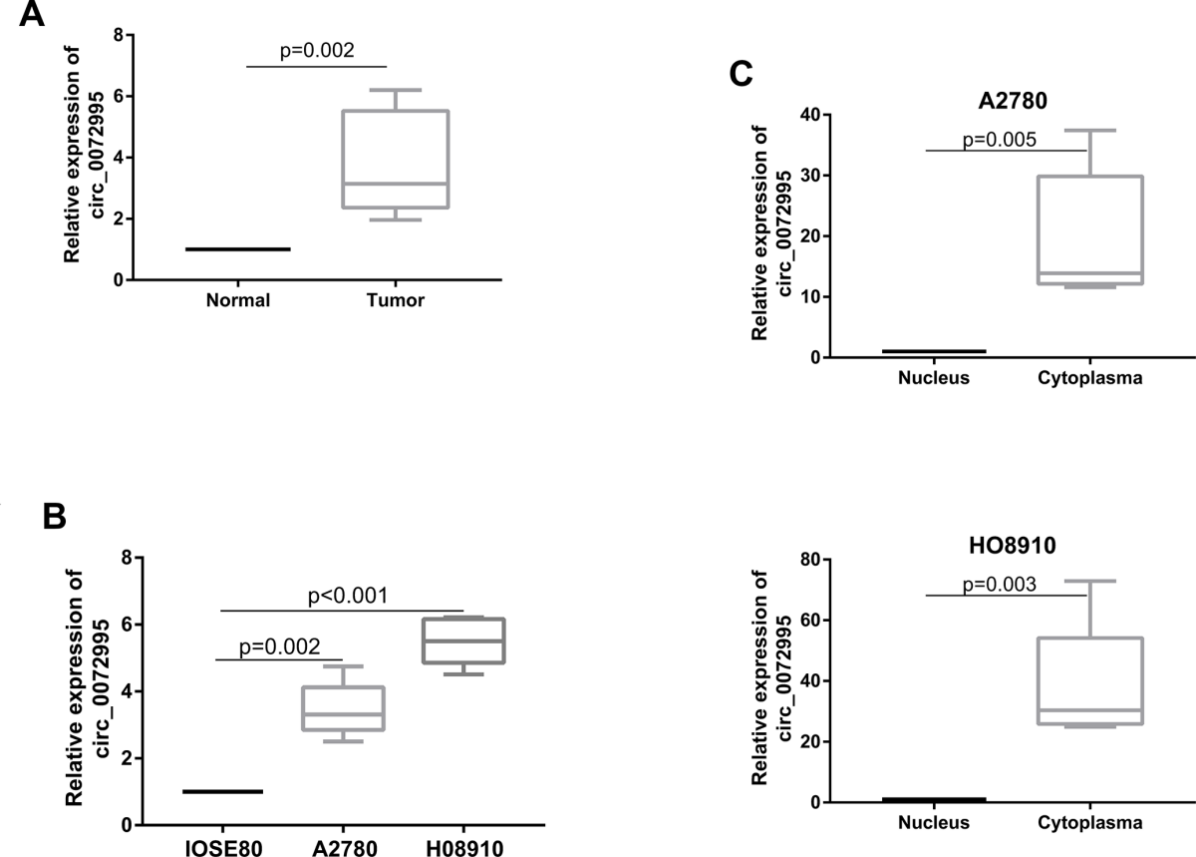
**circ_0072995 upregulated in EOC tissue and cell lines.** (**A**) circ_0072995 expression upregulated in EOC tissue compared with normal ovarian tissues as measured by qRT-PCR. (**B**) circ_0072995 expression upregulated in EOC cells compared with normal ovarian cells as measured by qRT-PCR. (**C**) circ_0072995 was mainly located in cytoplasm.

We found significantly higher expression of circ_0072995 in EOC afflicted tissues, compared with normal ovarian epithelial tissues (Figure 1A). Circ_0072995 was remarkably overexpressed in EOC cell lines (HO8910 and A2780) in comparison to normal ovarian cell line IOSE80 (Figure 1B). These data suggested and helped to confirm the potential for a relationship between circ_0072995 and EOC. Next, expression of circ_0072995 was analyzed in 40 EOC patients. To further clarify relationships between expression of circ_0072995 and the clinicopathological characteristics of EOC, tumor samples were divided into low and high circ_0072995 expression groups. As shown in Table 1, high levels of expression of circ_0072995 were positively correlated with pathological grades. In contrast, other parameters including such as age, menopausal stage, and FIGO stage shared no significant correlations with the levels of expression of circ_0072995. Taken together, these results indicated that there was a vitally important relationship between circ_0072995 and EOC. Importantly, qRT-PCR results for cytoplasmic and nuclear fractions revealed that circ_0072995 was mainly located in the cytoplasm (Figure 1C). In our view, this suggested that circ_0072995 may have executed its functions in the cytoplasm.

**Table 1:** Correlation between circ_0072995 expression and clinicopathological features in 40 EOC patients (cohort 1).

**Characteristics**	**Circ_0072995**		**P value**
	**Low (20)**	**High (20)**	
**AGE**			0.342
≥60	8	11	
<60	12	9	
**menopausal**			0.744
Premenopausal	7	8	
Postmenopausal	13	12	
**Pathological stage**			0.026
Low	6	13	
High	14	7	
**FIGO Stage**			0.209
I	7	5	
II	5	2	
III	8	13	
IV	0	0	

### Knockdown of circ_0072995 suppressed EOC cell proliferation and migration, induces apoptosis in vitro

**Figure 2:**
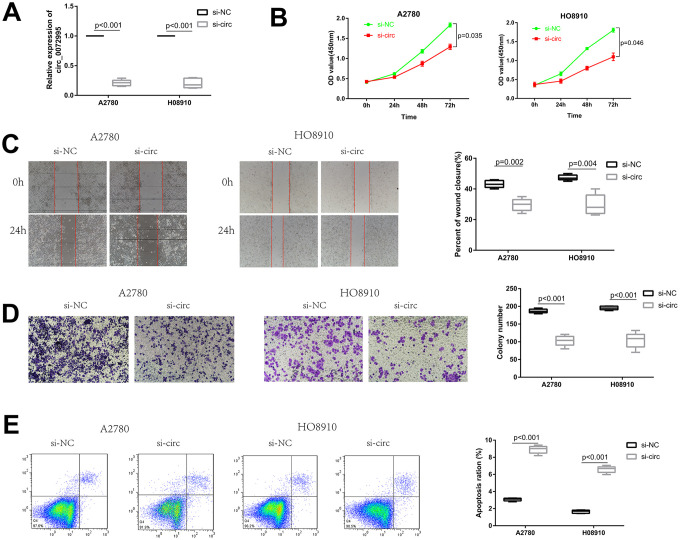
**Knockdown of circ_0072995 suppressed EOC cell proliferation and migration, induces apoptosis in vitro.** (**A**) Transfection efficiency of circ_0072995 knocking down determined by qRT-PCR. (**B**) Effect of circ_0072995 and si-circ_0072995 upon EOC cell growth curves as detected by CCK-8 assays. (**C**, **D**) The effect of circ_0072995 and si-circ_0072995 on EOC cell migratory and invasive capabilities as detected by wound healing and transwell assays. (**E**) The effect of circ_0072995 and si-circ_0072995 on EOC cell apoptosis as detected by flow cytometry apoptosis.

To explore the biological function of circ_0072995 in EOC cells, a RNAi vector against circ_0072995 was designed to silence circ_0072995 (Figure 2A). Knockdown of circ_0072995 significantly reduced the proliferation of HO8910 and A2780 cells, as confirmed by CCK-8 assays (Figure 2B). These results indicated that as an oncogene, circ_0072995 can enhance the proliferation of EOC cells. Then, to facilitate exploration of the effects of circ_0072995 on EOC cell migration and invasion, we performed wound healing and transwell analyses. These experiments indicated that after knockdown of circ_0072995, migration and invasion abilities of HO8910 and A2780 cells were significantly suppressed (Figure 2C, 2D). We recorded increased apoptosis in HO8910 and A2780 cells post-knockdown of circ_0072995, as demonstrated by flow cytometry analysis with Annexin V/PI double staining (Figure 2E). These data indicated that knocking down of circ_0072995 inhibited EOC cell proliferation and migration, and induced apoptosis in vitro.

### circ_0072995 functions as a sponge for miR-147a

**Figure 3:**
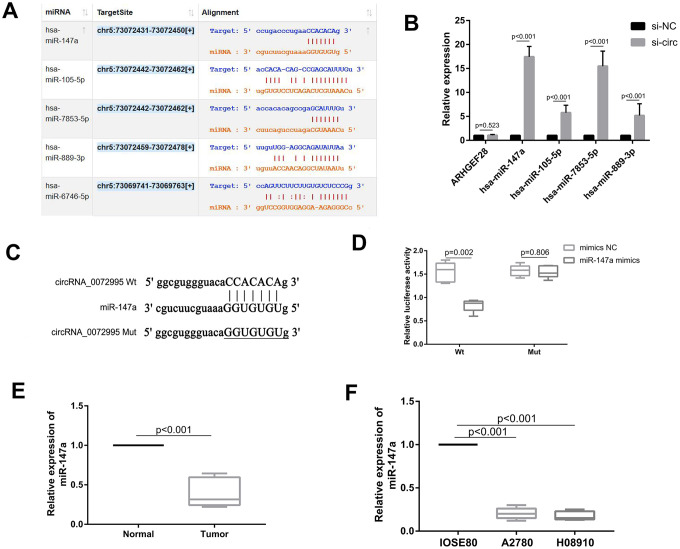
**circ_0072995 functioned as a sponge for miR-147a.** (**A**) Potential targets of circ_0072995 as predicted by Starbase software and according to seed-matching sequence. (**B**) miR-147a was selected as the target of circ_0072995 due to its expression and was significantly increased among the four miRNAs after knockdown of circ_0072995. (**C**) The potential binding sites between miR-147a and circ_0072995. (**D**) The binding relationship between miR-147a and circ_0072995 was confirmed by dual-luciferase reporter assays. (**E**, **F**) miR-147a expression was markedly downregulated in EOC afflicted tissues and cell lines compared with normal groups, as determined by qRT-PCR.

To explore underlying molecular mechanisms of circ_0072995, we focused upon potential circ_0072995-binding miRNAs because cytoplasmic circRNA works mainly via the ceRNA mechanism [12]. Firstly, we predicted the potential downstream target genes of circ_0072995 with circRNA target prediction software Starbase [18] (Figure 3A and Supplementary File 1). The top four miRNAs were selected for the next stages of the experiment. Additionally, we found that knocking down of circ_0072995 significantly increased expression levels of the four miRNAs in HO8910 cells, especially with respect to miR-147a (Figure 3B). Meanwhile, we also found that knocking down of circ_0072995 had no effect on its host gene ARHGEF28 as showed in Figure 3B. Next, the potential binding sites of circ_0072995 and miR-147a were determined using computational prediction approaches in Starbase software (Figure 3C). The binding ability between circ_0072995 and miR-147a was confirmed by luciferase reporter assays. The full-lengths of circ_0072995-WT and of the mutant version without miR-147a binding sites were subcloned into luciferase reporter vector psiCHECK2. The results indicated that miR-147a mimics significantly decreased the luciferase activity of the WT group but did not have this effect upon the mutant treatment group (Figure 3D). Next, the levels of expression of miR-147a were detected in EOC afflicted tissues and EOC afflicted cell lines. The results indicated that miR-147a was markedly down regulated in EOC afflicted tissues and cell lines compared with normal groups (Figure 3E, 3F). Collectively, these data suggested a possible direct interaction between circ_0072995 and miR-147a, and demonstrated that circ_0072995 could act as a sponge of miR-147a in EOC.

### Overexpression of miR-147a inhibits EOC cell proliferation and migration, induces apoptosis in vitro

**Figure 4:**
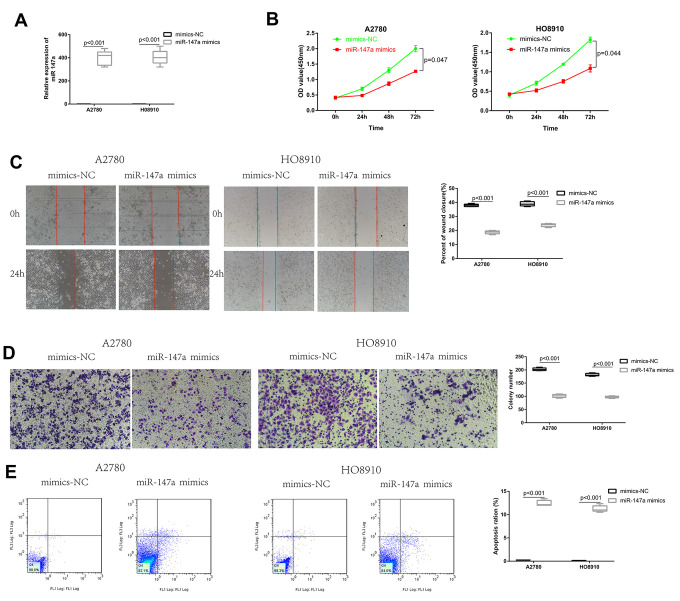
**Overexpression of miR-147a inhibited EOC cell proliferation and migration, induced apoptosis in vitro.** (**A**) Transfection efficiency of miR-147a mimics was determined by qRT-PCR. (**B**) The effect of miR-147a and miR-147a mimics on EOC cell growth curves was detected by CCK-8 assays. (**C**, **D**) The effect of miR-147a and miR-147a mimics on EOC cell migratory and invasive capabilities was detected by wound healing and transwell assays. (**E**) The effect of miR-147a and miR-147a mimics on EOC cell apoptosis was detected by flow cytometry apoptosis.

To explore the biological function of miR-147a in EOC cells, miR-147a mimics and inhibitor were constructed. Transfection efficiency of miR-147a mimics was determined by qRT-PCR (Figure 4A). We found that upregulation of miR-147a significantly suppressed by way of the ability of HO8910 and A2780 cells to proliferate, as confirmed by CCK-8 assays (Figure 4B). These results suggested that miR-147a suppressed proliferation of EOC cells. Then, to explore the effects of miR-147a upon migration and invasion of EOC cells, we performed wound healing and transwell analyses. These experiments indicated that after inducing overexpression of miR-147a, the migration and invasion abilities of HO8910 and A2780 cells were significantly suppressed (Figure 4C, 4D). There was also increased apoptosis in HO8910 and A2780 cells and overexpression of miR-147a, as demonstrated by flow cytometry analysis with Annexin V/PI double staining (Figure 4E). Ultimately, these data indicated that overexpression of miR-147a inhibited EOC cell proliferation and migration and induced apoptosis.

### CDK6 is directly targeted by miR-147a and indirectly regulated by circ_0072995

**Figure 5:**
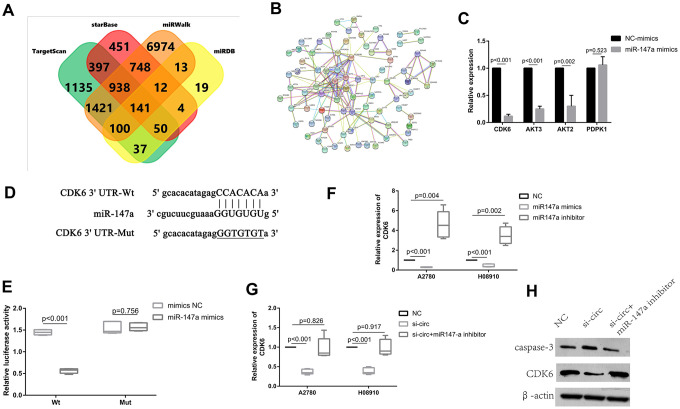
**CDK6 was directly targeted by miR-147a and was indirectly regulated by circ_0072995.** (**A**) The target genes of miR-147a were predicted in databases (TargetScan, miRWalk, miRDB, StarBase) and there were 141 genes which overlapped in the Venn diagram. (**B**) PPI network analyses was performed by use of the String database, and results with a combined score of > 0.4 were selected. Among these, CDK6, AKT3, AKT2, PDPK1 were found to have had a high degree of connectivity and were selected as hub genes for miR-147a. (**C**) CDK6, AKT3, AKT2 expression was significantly reduced after overexpression of miR-147a, especially for CDK6. (**D**) There were multiple binding sites between miR-147a and CDK6. (**E**) The binding relationship between miR-147a and CDK6 was confirmed by dual-luciferase reporter assays. (**F**) miR-147a mimics markedly reduced expression of CDK6, whereas miR-147a inhibitors significantly enhanced CDK6 levels in HO8910 and A2780 cells. (**G**) Expression of CDK6 mRNA decreased after knockdown of circ_0072995, but was rescued by miR-147a inhibitors as determined by qRT-PCR. (**H**) The expression of CDK6 proteins decreased after knockdown of circ_0072995, but was rescued by miR-147a inhibitors as determined by WB.

The target genes of miR-147a were predicted from the databases (i.e., TargetScan, miRWalk, miRDB, and StarBase) according to the seed-matching sequence. After entering miR-147a in search boxes for these websites, we completed and exported the results for the target gene of miR-147a. There were 4219 genes resultant from TargetScan, 10346 genes from miRWalk, 374 genes from miRDB, and 2741 genes from StarBase. There were 141 genes that remained in the overlapped spaces a in a Venn diagram (Figure 5A and Supplementary File 2). The STRING tool (https://string-db.org/) was used to obtain PPI relationships for the 141 genes. PPIs with a combined score of > 0.4 were then selected. Among these, CDK6, AKT3, AKT2, PDPK1 demonstrated a high degree of connectivity and were subsequently selected as the hub genes for miR-147a (Figure 5B). After inducing overexpression of miR-147a, CDK6 expression was significantly reduced, and there were resultant multiple binding sites between them (Figure 5C, 5D). Therefore, we then next selected CDK6 for subsequent experiments and dual luciferase reporter assays performed to confirm this prediction. Results indicated that miR-147a mimics significantly reduced the activity of the luciferase reporter vector carrying the CDK6 3’UTR-WT sequence instead of carrying the mutant sequence (Figure 5E). Moreover, miR-147a mimics significantly suppressed levels of CDK6, whereas miR-147a inhibitors markedly enhanced expression of CDK6 in HO8910 and A2780 cells (Figure 5F). To investigate if circ_0072995 regulated expression of CDK6 in EOC cells, the induction of downregulation of circ_0072995 was used and resulted in significantly decreased expression of CDK6. However, these effects were rescued by use of miR-147a inhibitor (Figure 5G, 5H). These data indicated that CDK6 was directly targeted by miR-147a and was indirectly regulated by circ_0072995. Lastly, we found that Circ_0072995 acted as a sponge of miR-147a such as to regulate the expression of CDK6 in EOC cells.

### Downregulation circ_0072995 suppressed tumor growth in nude mice xenografts

**Figure 6:**
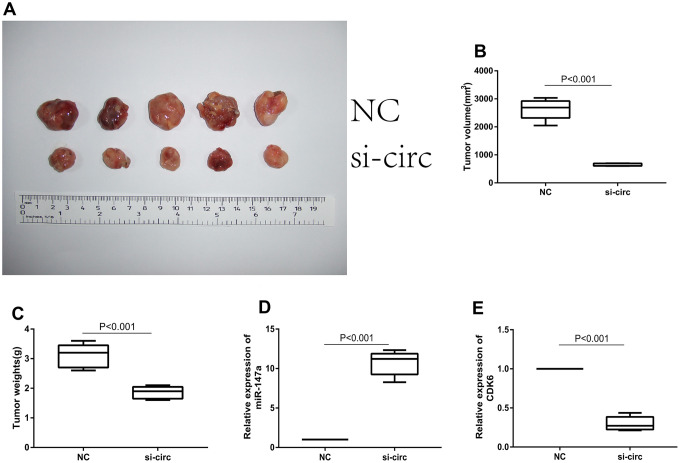
**Downregulation of circ_0072995 suppressed tumor growth in nude mice xenografts.** (**A**) Representative images of tumor formation in xenografts of nude mice. (**B**) Tumor weight was suppressed after downregulation of circ_0072995 as measured 30 days post-injection. (**C**) Tumor volume was suppressed after downregulation of circ_0072995 as measured 30 days post-injection. (**D**) Expression of miR-147a was enhanced after downregulation circ_0072995 as determined by qRT-PCR. (**E**) The expression of CDK6 was suppressed after downregulation circ_0072995 as determined by qRT-PCR.

**Figure 7:**
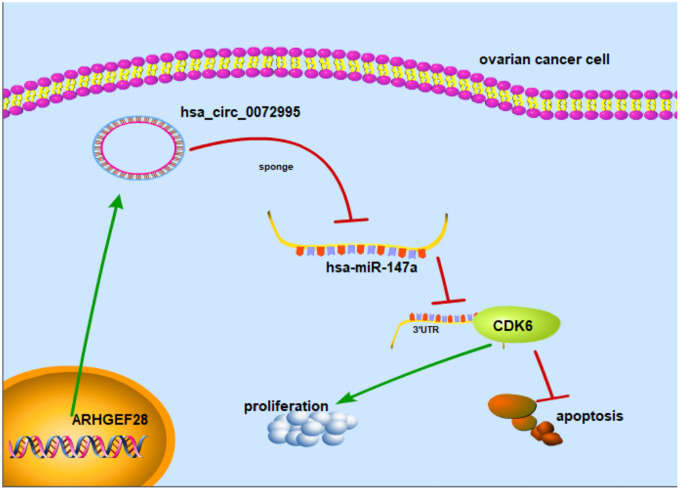
**Mechanism diagram.** Schematic diagram indicates that circ_0072995 promoted tumorigenesis and progression in EOC afflicted cells.

To explore whether or not circ_0072995 played a role in the progression of ovarian cancer in vivo, lentiviral stable strains of circ_0072995 knockdown HO8910 cells were constructed and were implanted into nude mice to induce subcutaneous tumor formation. We found, that compared with the lentiviral-stabilized negative control (NC) group, knockdown of circ_0072995 suppressed tumor growth in both weight and volume (Figure 6A–6C). Therefore, we concluded that down-regulation of circ_0072995 prevented tumor formation in nude mice xenografts. Moreover, qRT-PCR detection revealed that silencing of circ_0072995 promoted miR-147a expression and suppressed the CDK6 expression (Figure 6D, 6E). These results indicated that circ_0072995 served in its role and through its function in the circ_0072995/miR-147a/CDK6 axis. In summary, these data illustrated that circ_0072995 might serve as a ceRNA for miR-147a to regulate CDK6 expression, that circ_0072995 caused abnormal proliferation of ovarian cancer cells, that circ_0072995 reduced apoptosis, and that circ_0072995 may have led to the occurrence and development of EOC (Figure 7).

## DISCUSSION

Owing to the development of bioinformatics and high-throughput sequencing in recent years, circRNAs have been increasingly examined in clinical experiments and have great potential as biomarkers for detection of disease [19, 20]. As a type of endogenous non-coding RNA, increasing evidence has revealed that circRNAs play crucial roles in multiple physiological and pathological related functions in cardiovascular disease [21], central nervous system afflictions [22], and in a large suite of types of cancers [20, 23]. For the most part and until only recently, only a few types of circRNAs have had been determined to influence dynamics underlying the progression of ovarian cancer, and the biological functions of most circRNAs have not been well described [24, 25].

In the present study, circ_0072995 was significantly upregulated in EOC afflicted tissues and cell lines, and was aberrantly correlated with EOC pathological grade. Thus, the findings suggested that circ_0072995 is related to the occurrence and development of ovarian cancer. However, whether or not circ_0072995 it related to survival of patients that are afflicted with ovarian cancer requires additional follow-up study and research.

Next, we performed functional experiments both in vivo, and in vitro and the findings indicated that downregulation of circ_0072995 suppressed proliferation and migration, as well as induced apoptosis of EOC afflicted cells. Furthermore, knockdown of circ_0072995 suppressed tumor growth in nude mice xenografts. These results suggest that circ_0072995 is an oncogene that plays an important role in the progression of EOC. Thus, we postulate that circ_0072995 may become a new and important diagnostic and prognostic marker for EOC and or a therapeutic target for afflicted patients.

CircRNAs consist of a circular loop with multiple miRNA binding sites; and have the common function of acting as sponges of miRNA in cytoplasm [12]. For instance, circFNDC3B directly bound to miR-1178-3p such as to inhibit bladder cancer progression [26], whereas hsa_circ_0018289 acted as a miR-497 sponge to promote cervical cancer progression [27]. Circ_0000190 possibly acted as a sponge of miR-767-5p such as to regulate the progression of multiple myeloma [28] and CircAGFG1 regulated the progression of TNBC by sponging miR-195-5p [29]. Based upon our approach, miR-147a was been predicted as a target of circ_0072995 using bioinformatics analyses and dual-luciferase reporter assays confirmed that circ_0072995 interacted with miR-147a directly. Further, our investigations suggested that miR-147a was significantly downregulated in EOC afflicted tissues and cell lines. Consistent with our results, it has also been reported that miR-147a was significantly downregulated in non-small-cell lung cancer patient tissues and cell lines and was negatively correlated with the degree of malignancy of non-small-cell lung cancer [30, 31]. To explore the regulatory mechanisms within circ_0072995 and miR-147a, we performed functional experiments and tested the interaction between them based upon progression of EOC cell lines. We found that miR-147a mimics significantly suppressed cell proliferation and migration of EOC cells, but induced apoptosis. This reflects that miR-147a can play a regulatory role in tumorigenesis as a tumor suppressor gene. Further, our findings suggested that miR-147a competitively combines with circ_0072995 and cannot exert an inhibitory effect, thus eventually leading to the progression of ovarian cancer.

CDK6, which is a member of the family of cyclin-dependent kinases, has been identified as a tumor oncogene that was upregulated and in a series of adenocarcinomas, including such as in breast cancer and non-small cell lung cancer [32, 33]. CDK6 direct-targeted miR-211, inhibited proliferation, and induced apoptosis of OC [34]. It has previously also been reported that CDK6 reduced the sensitivity of EOC cells to platinum [35]. In our assessments, CDK6 was predicted as a target of miR-147a by using bioinformatics, and this prediction was validated by luciferase reporter assays. Inducing reduced expression of miR-147a consequently remarkably enhanced the levels of CDK6, whereby inducing increased expression of miR-147a achieved the opposite effect upon CDK6 expression. These findings demonstrated that CDK6 was a direct target of miR-147a. In our research we also found that knocking down of circ_0072995 significantly suppressed the levels of expression of CDK6, and that this effect could be reversed by the addition of miR-147a inhibitors. These findings revealed that circ_0072995 exerted influence as an oncogene in EOC afflicted cells via negatively targeting miR-147a. Mechanistically, circ_0072995 functioned as miR-147a sponges removing the inhibitory effect of miR-147a on its target CDK6, and further regulated the expression of CDK6. Ultimately, our research suggested that circ_0072995 is a good candidate for considerations aimed at improving the diagnosis of EOC, and treatment outcomes for patients afflicted by EOC.

## CONCLUSIONS

In summary, circ_0072995 is an oncogene that appears to play crucial roles in the progression of EOC. Upregulation of circ_0072995 effectively enhanced cell proliferation and invasion in vitro and promoted tumor growth in vivo by way of targeting the miR-147a/CDK6 axis. The increased understanding the regulatory mechanisms of circ_0072995, which we described herein, could lead a better understanding of novel and improved strategies and treatment outcomes for EOC afflicted patients.

## MATERIALS AND METHODS

### Patients and samples collection

The 40 pairs of EOC samples and normal ovarian samples were obtained from patients who were diagnosed with EOC at the Second Affiliated Hospital of Harbin Medical University (Harbin, China). We excluded patients with other malignant types of tumors, and excluded those that did not receive preoperative chemotherapy or radiotherapy. Tissues were collected after surgical resection and were stored in liquid nitrogen until further use. The diagnosis, and determinations of stage and risk status of EOC were made in accordance with the International Federation of Gynecology and Obstetrics (FIGO). Written informed consent was obtained from all patients prior participation in this study. The present study was authorized by the Ethics Committee of The Second Affiliated Hospital of Harbin Medical University.

### Cell culture and transfection

The human EOC cell lines HO8910, A2780, and the human normal ovarian epithelial cell line IOSE80 were cultured in DMEM (Gibco Gaithersburg, MD) containing 10 % fetal bovine serum (FBS, Gibco). The above cell lines were cultured at 37 °C in an atmosphere of 5 % CO2. Primer sequences were as follows: hsa_circ_0072995 (5’- AGAACAGCTATGCCCTCCAG-3’, 3’-CCCATCTCATAGCCAGGTGT-5’), hsa-miR-147a (5’-GCGGGCGTGTGTGAAATGC-3’, 3’-ATCCAGTGCAGGGTCCGAGG-5’), and CDK6 (5’CCGTGGATCTCTGGAGTGTT3’, 3’GGTTGGGCAGATTTTGAATG5’). Small interfering RNAs for circRNA 0072995 (si-circRNA: 5’-CAGAAACTGAAGAAGTATC-3’), miR-147a mimics (5’-GUGUGUGGAAAUGCUUCUGC-3’), miR-147a inhibitors (5’-mGmCmAmGmAmAmGmCmAmUmUmUmCmCmAmCmAmCmAmC-3’), and negative controls were purchased from RIBOBIO Company (GuangZhou, China). We transfected into above cell lines using Lipofectamine 3000. Circ_0072995 silenced lentiviral stabilized HO8910 cells were constructed (sh-circ_0072995) prior to the initiation of in vivo experiments.

### Bioinformatics predictions

The web-based online tool StarBase (V3 version, http://starbase.sysu.edu.cn/index.php) was used for targeting between circRNA and miRNA prediction. The online Starbase, Targetscan (7.2 version, http://www.targetscan.org/vert_71/), miRWalk (http://mirwalk.umm.uni-heidelberg.de/), miRDB (http://mirdb.org/) were used for prediction of the targets miR-147a. The protein-protein interaction (PPI) network was constructed by the STRING database (11.0 version, https://string-db.org/).

### q-PCR

Total RNA was extracted with TRIzol reagent (Invitrogen). Reverse transcription was then performed with a complementary DNA (cDNA) synthesis kit (Aidlab, Beijing, China). Subsequently, we subjected cDNA to quantitative reverse transcription polymerase chain reaction (qRT-PCR) on an ABI 7900HT sequencer (Thermo Fisher Scientific, Waltham, MA) with a SYBR Green qPCR Mix (Aidlab). All experiments were performed in duplicate. The 2^-ΔΔCt^ method was used to calculate relative expression.

### Western blotting

Twenty micrograms of extracted and calibrated protein was separated by sodium lauryl sulfate-polyacrylamide gel electrophoresis and was then transferred to a nitrocellulose membrane (Millipore, Madison, WI). We then incubated with primary antibodies at 4 °C overnight, and incubated the membrane with a horseradish peroxidase-conjugated secondary antibody. Signals were detected using Immobilon ECL substrate (Millipore, Germany), and images were acquired by use of an Optimax X-ray Film Processor (Protec, Germany). β-actin was used as an internal control. CDK6, caspase3, and β-actin antibodies were purchased from Affinity Biosciences (Affinity Biosciences, China).

### Nuclear-cytoplasmic fractionation

Cytoplasmic and nuclear RNAs were isolated using NE-PER Nuclear and Cytoplasmic Extraction Reagents (Thermo Scientific, USA) following all manufacturer protocols. We followed this up with qRT-PCR.

### Cell proliferation and apoptosis assays

Cell proliferation was examined using Cell Counting Kit 8 (Invitrogen, Carlsbad, CA). That is, 100 μL of cell suspension (2,000 cells • well-1) were seeded into a 96-well plate. Next, after 0, 24, 48, and 72 hours (h) of culture, we added 10 μL of Cell Counting Kit 8 reagent and incubated for 1 h prior to making detections. Cell viabilities were examined by measuring optical density at 450 nm with a specrophotometric plate reader (BioTek, VT, USA). Each experiment was repeated in triplicate.

For apoptosis assays, HO8910 or A2780 cells were harvested after transfection and double stained using fluoresce in isothiocyanate (FITC)-conjugated Annexin V and propodium iodide (PI). Next, the percentage of early apoptotic cells was analyzed on a flow cytometer (Becon Dickinson FACSCalibur, NY, USA). Apoptosis was examined by One Step TUNEL (TdT-mediated dUTP Nick-End Labeling) Apoptosis Assay Kit (Beyotime, Shanghai, China) in accordance with manufacturer protocols. HO8910 or A2780 cells were fixed with 4 % paraform for about 30 minutes (min), then stained with Hoechst 33342 (Beyotime, Shanghai, China) for 20 min and photographed under light fluorescence microscopy (Leica, Wetzlar, Germany). We performed three independent replicated experiments for these analyses.

### Wound healing and migration assay

HO8910 or A2780 cells were seeded in 6-well plates and scratched with a 200 μL sized pipette tip in the well centers at 24 h post-transfection. We then cultured the cells in serum-free medium. After 24 h, wound widths were recorded at three-independent wound sites per group and were normalized to the control group.

For migration analyses, a pipette chamber containing 24 microwells and an 8 μm membrane (BD Biosciences, Franklin Lakes, NJ) were used. HO8910 or A2780 cells from different groups were placed into the upper part of the chamber at a density of 200 μL. We removed the serum-free medium while inserting 500 μL of complete medium into the bottom chamber. After 24 h of incubation, the cells at the bottom of the chamber were then fixed, counted with 4 % paraformaldehyde, and stained with crystal violet.

### Dual-luciferase reporter assay

Reporter plasmids were constructed by adding wild/mutation circRNA or CDK6 3’-UTR sequence to the pGL3 vector (Promega, Madison, WI). Lipofectamine 2000 was then used and miR-147a mimics combined with reporter plasmids were cotransfected into 239T cells. We used the dual-Luciferase Reporter Assay System (Promega, Sunnyvale, CA) to detect firefly and Renilla luciferase activities after culturing for 48 h following all manufacturer protocols.

### Animal experiments

For the animal study, 12 BALB/c nude mice at ages of approximately 4 to 6 weeks old and weighing approximately 15 to 20 g were obtained from Shanghai Laboratory, Animal Research Center (Shanghai, China). The Ethics Committee of The Second Affiliated Hospital of Harbin Medical University approved all animal experiments in this study. HO8910 cells (2×10^7^) with or without circ0072995 silencing were injected into the right flank of nude mice. Vernier calipers were used for tumor size measurements used to calculate tumor volume (volume = 1/2×length×width^2^).

### Statistical analyses

We used GraphPad Prism software (GraphPad, La Jolla) for data analyses. Chi-square tests were completed in SPSS (Version 17.0 Chicago, USA). Data were calculated as the mean ± SEM. A P ≤ .05 indicated a level of statistical significance at which the null hypothesis of no differences among treatment groups would be rejected.

### Availability of data and materials

All data generated or analyzed during this study are included in this published article.

### Ethics approval and consent to participate

The present study was approved by the Ethics Committee of the The Second Affiliated Hospital of Harbin Medical University. All procedures performed in studies involving animals were in accordance with the ethical standards of the institution or practice at which the studies were conducted.

### Patient consent for publication

All procedures performed in studies involving human participants were in accordance with the ethical standards of the institutional and/or national research committee and with the 1964 Helsinki declaration and its later amendments or comparable ethical standards.

## Supplementary Material

Supplementary File 1

Supplementary File 2
